# Fluoroscopy-Free Pulmonary Vein Isolation in Patients with Atrial Fibrillation and a Patent Foramen Ovale Using Solely an Electroanatomic Mapping System

**DOI:** 10.1371/journal.pone.0148059

**Published:** 2016-01-28

**Authors:** Michael Kühne, Sven Knecht, Aline Mühl, Tobias Reichlin, Nikola Pavlović, Arnheid Kessel-Schaefer, Beat A. Kaufmann, Beat Schaer, Christian Sticherling, Stefan Osswald

**Affiliations:** 1 Cardiology/Electrophysiology, University Hospital Basel, Basel, Switzerland; 2 University Hospital Centre ''Sisters of mercy'', Zagreb, Croatia; University of Minnesota, UNITED STATES

## Abstract

**Introduction:**

The advent of electroanatomical mapping (EAM) systems for pulmonary vein isolation (PVI) has dramatically decreased radiation exposure. However, the need for some fluoroscopy remains for obtaining left atrial (LA) access. The aim was to test the feasibility of fluoroscopy-free PVI in patients with atrial fibrillation (AF) and a patent foramen ovale (PFO) guided solely by an EAM system.

**Methods:**

Consecutive patients with AF undergoing PVI and documented PFO were studied. An EAM-guided approach without fluoroscopy and ultrasound was used. After completing the map of the right atrium, the superior vena cava and the coronary sinus, a catheter pull-down to the PFO was performed allowing LA access. The map of the LA and subsequent PVI was also performed without fluoroscopy.

**Results:**

30 patients [age 61±12 years, 73% male, ejection fraction 0.64 (0.53–0.65), LA size in parasternal long axis 38±7 mm] undergoing PVI were included. The time required for right atrial mapping including transseptal crossing was 9±4 minutes. Total procedure time was 127±37 minutes. Fluoroscopy-free PVI was feasible in 26/30 (87%) patients. In four patients, fluoroscopy was needed to access (n = 3) or to re-access (n = 1) the LA. In these four patients, total fluoroscopy time was 5±3 min and the DAP was 14.9±13.4 Gy*cm^2^. Single-procedure success rate was 80% (24/30) after a median follow-up of 12 months.

**Conclusion:**

In patients with a documented PFO, completely fluoroscopy-free PVI is feasible in the vast majority of cases.

## Introduction

The U.S. Nuclear Regulatory Commission recommends making every effort to keep exposure to ionizing radiation as low as reasonably achievable (ALARA), a statement that is endorsed by the major societies of physicians working with radiation[1]. Pulmonary vein isolation (PVI) is the mainstay of interventional treatment in patients with atrial fibrillation (AF) [2]. PVI was initially performed mainly under fluoroscopic guidance, but the accuracy and novel features of latest-generation electroanatomic mapping (EAM) systems have led to a dramatic decrease in radiation exposure over the recent years [3].

Patent foramen ovale (PFO) is a congenital condition allowing access from the right atrium (RA) to the left atrium (LA) without transseptal puncture. The prevalence of PFO is approximately 25% in the general population based on probe-patency in autopsy studies and on transesophageal echocardiography (TEE) [4,5].

The purpose of this study was to evaluate the feasibility of completely fluoroscopy-free PVI using a state-of-the-art EAM system in patients with atrial fibrillation and a patent foramen ovale (PFO) without any additional technical equipment such as peri-procedural intracardiac or transesophageal echocardiography.

## Methods

All patients undergoing PVI were screened during the routine pre-procedural transesophageal echocardiography, and in case of the presence of a PFO, were included in the study. Exclusion criteria for the study were a history of percutaneous or surgical PFO closure and the presence of mechanical mitral or tricuspid valves. The study was approved by the Ethics Committee Northwest Switzerland (EKNZ) on human research and conducted according to the principles expressed in the Declaration of Helsinki. Written informed consent was obtained from all patients.

### Echocardiography

**Fig 1:**
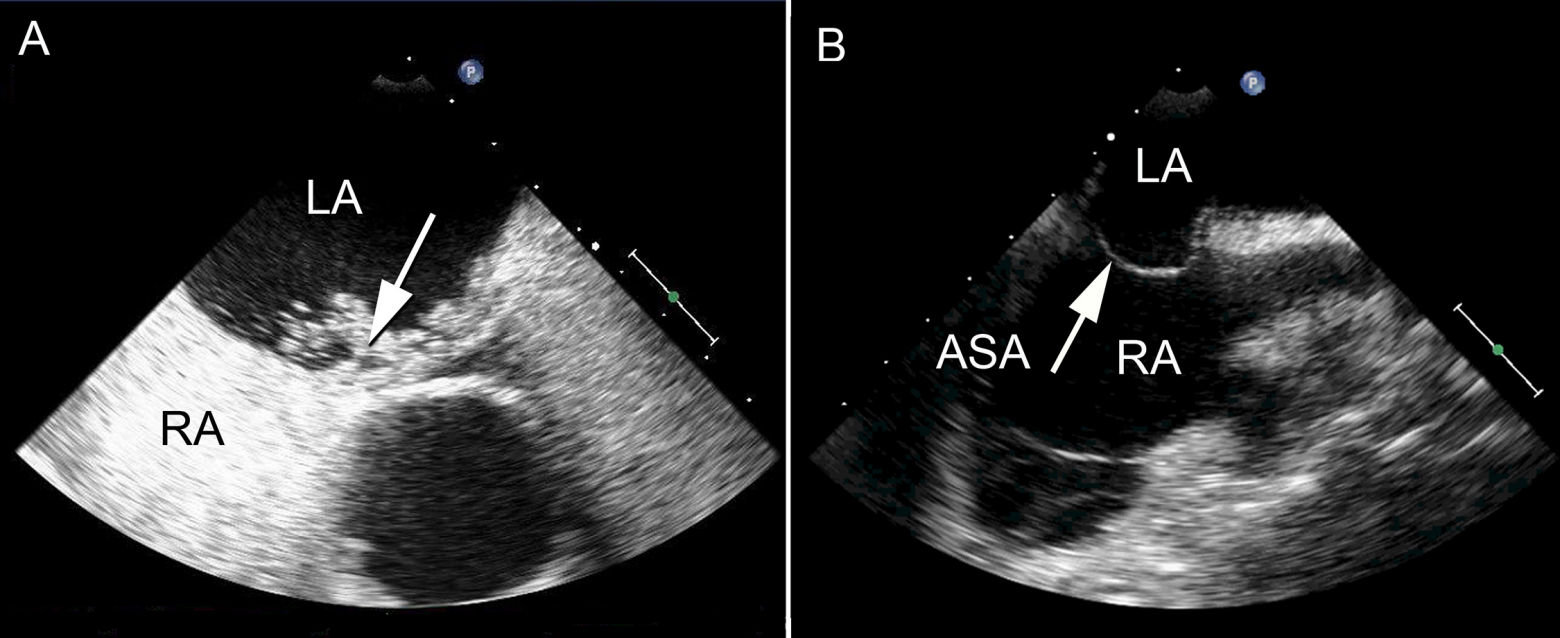
(A) Pre-procedural transesophageal echocardiography showing a medium-sized PFO (arrow) based on the extent of right-to-left shunt of agitated colloidal contrast. (B) Transesophageal echocardiography of a patient with a PFO and an atrial septal aneurysm (ASA; excursion of the septum of more than 15mm throughout the cardiorespiratory cycle). LA = left atrium; RA = right atrium.

All patients underwent transesophageal echocardiography (under propofol sedation) to rule out thrombus in the left atrium (LA) and to assess the anatomy of the interatrial septum before the procedure. The septum was classified as 1) “normal”, 2) “hypermobile”, (excursion of the septum of 10 up to 15 mm), or 3) “atrial septal aneurysm” (excursion of the septum of more than 15mm throughout the cardiorespiratory cycle). The presence of a PFO was determined after intravenous injection of a bolus of 5 ml of agitated colloidal contrast in all patients. PFOs were semi-quantitatively classified as “small”, “medium”, or “large” according to the extent of right-to-left shunt of contrast (Fig 1) [6].

### Magnetic resonance imaging

Magnetic resonance imaging (MRI) was performed as previously described on a 1.5T scanner (MagnetomAvanto/ Espree, Siemens, Germany) equipped with phased array body coils [7]. Routine multiplanar scout images were acquired. A respiratory- and ECG-gated 3 dimensional balanced steady state free precession (bSSFP) sequence was acquired in axial orientation covering the LA. A navigator was placed perpendicular to the liver/lung interface enabling breath-gating during free breathing. Subsequently, a 3D contrast enhanced Magnetic Resonance Angiography was acquired.

### Electrophysiological procedure

Indications for PVI were based on current guidelines [8]. Oral anticoagulation was not interrupted for the procedure in patients on Vitamin-K antagonists. In patients on dabigatran or rivaroxaban, the dose was held the morning of the procedure.

PVI was performed under conscious sedation using midazolam, fentanyl, and propofol. Intravenous heparin was used to maintain an activated clotting time of 350 seconds. The sheaths were continuously flushed with heparinized saline. Intracardiac electrograms and surface electrograms were recorded and displayed at a speed of 100 mm/s. The procedural endpoint was the elimination of all PV potentials on the 20-pole circular mapping catheter. All procedures were performed in conjunction with a 3D EAM system (Carto3, Biosense Webster, Diamond Bar, CA, USA).

In all patients, an approach using the EAM system only was attempted with the goal not to use fluoroscopy (nor any other additional technology such as echocardiography). However, fluoroscopy could be used at the operator’s discretion at any time.

### Mapping and ablation

**Fig 2:**
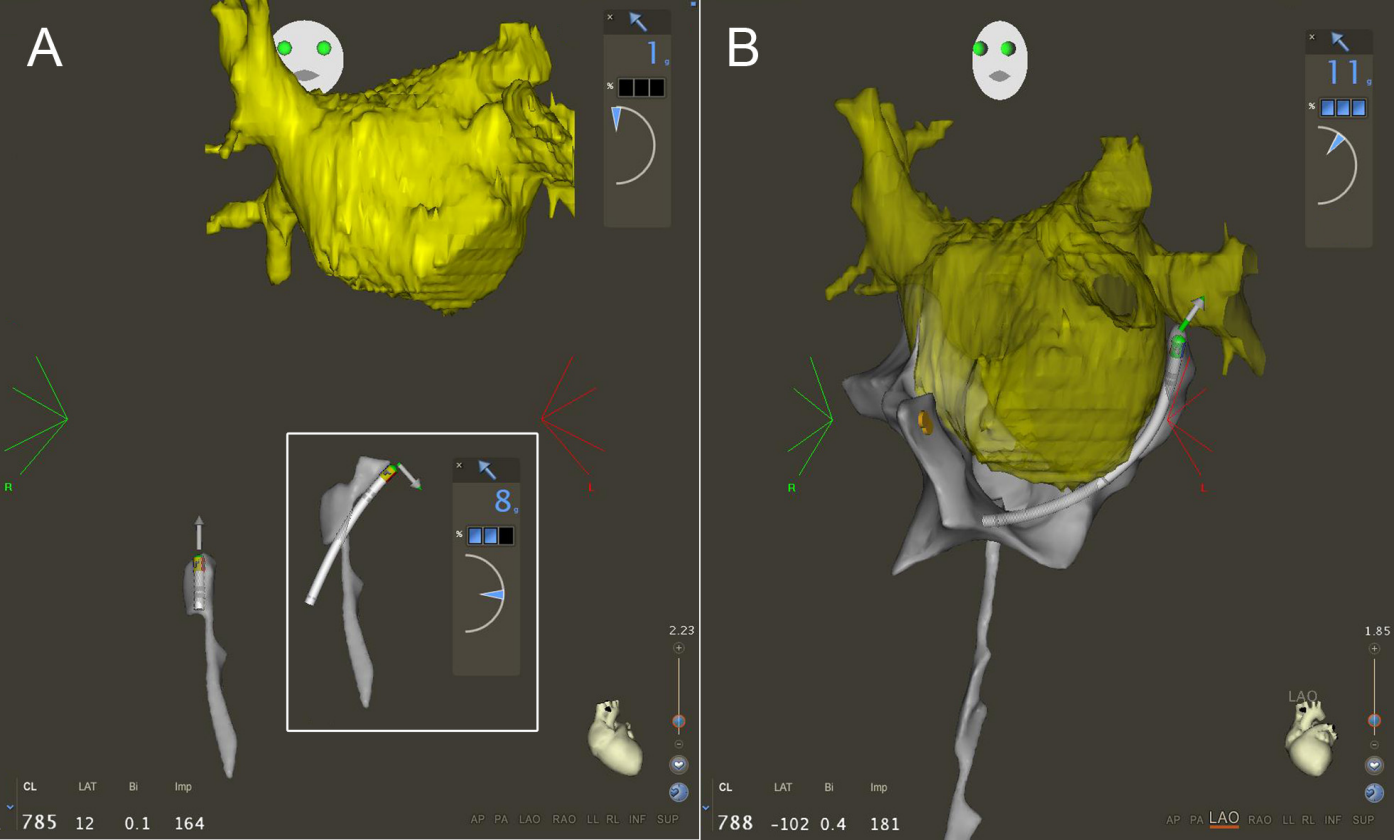
(A) Antero-posterior view of the fast anatomical map of the inferior vena cava. While advancing the catheter up to the right atrium, catheter tip orientation, force vector orientation, and contact force are monitored (outlined box). (B) Left anterior oblique view of the map of the right atrium with the 3D MRI reconstruction of the left atrium positioned based on the course of the coronary sinus and the septum. The yellow tag denotes the His position.

In all patients, a pre-procedural 3D MRI reconstruction of the LA was performed using the CartoMerge (Biosense Webster) Software Module and was imported into the Carto3 system. The ablation catheter (Thermocool, Biosense Webster) was connected to the Carto3 system before it was inserted into the body. After obtaining vascular access via the right femoral vein, a transseptal sheath was advanced to the right external or common iliac vein over a long guidewire. Then, the ablation catheter was slowly advanced to the RA, avoiding any unusual resistance while advancing the catheter. As soon as visualization was available, catheter orientation was monitored on the EAM system. In cases where a contact force-sensing catheter (Thermocool Smart Touch, Biosense Webster) was used, the contact force information and vector orientation was used when advancing the catheter up to the heart (Fig 2A). As soon as available, the fast anatomical mapping (FAM) feature of Carto3 was started and used to map the course of the inferior vena cava (IVC). The intracardiac electrogram on the catheter tip was monitored until atrial signals were recorded. The earliest point, where near-field atrial electrograms were recorded was marked on the map as the entrance of the IVC into the RA. The catheter was then advanced into the superior vena cava (SVC), and the SVC-right atrial junction was marked as the point where atrial signals on the catheter tip were lost again.

**Fig 3:**
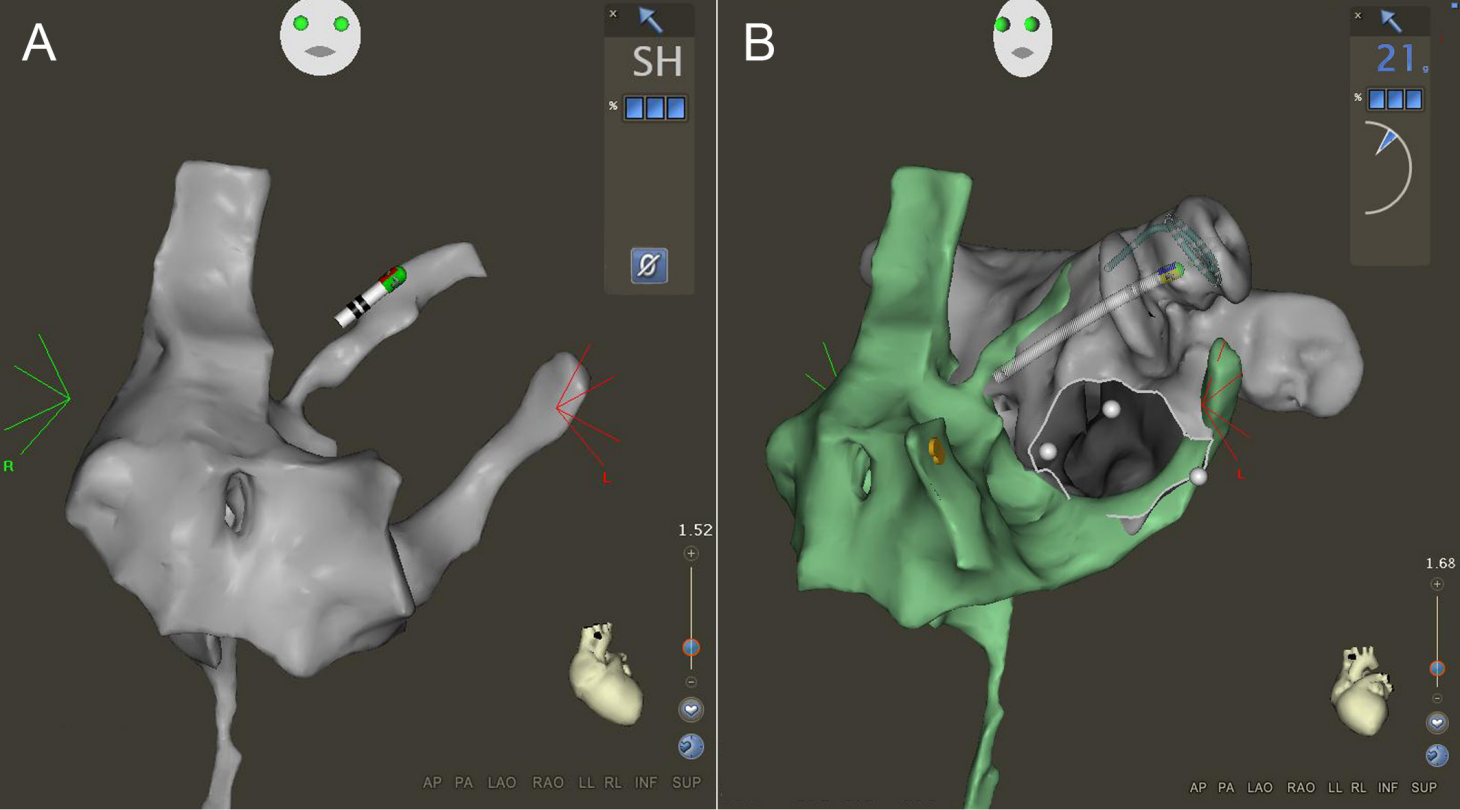
(A) Anterior-posterior view of the right atrial map including the path of the ablation catheter into the left atrium. Note that the color of the proximal ring electrodes of the ablation catheter have switched from the normal grey to black indicating their position within the sheath, thereby ensuring left atrial position of the transseptal sheath. (B) The ablation catheter is then exchanged for the circular mapping catheter to obtain a map of the left atrium. The ablation catheter is then inserted into the second transseptal sheath and left atrial access is gained based on the known location and orientation of the PFO. The white line and the three white tags delineate the mitral annulus. Note that the right atrial map is switched to a different color (green) to clearly appreciate the edges of the two maps.

Once the map of the SVC-IVC axis was completed, the RA was mapped using right and left anterior oblique views in the Carto3 system by advancing the catheter into the SVC and pulling it into the IVC in different axial rotations with a slight deflection. After mapping the RA and tagging the His position, the catheter was deflected and introduced into the coronary sinus (CS) by clockwise rotation and advanced to a left lateral position. The MRI reconstruction of the LA was then positioned based on the course of the CS and the septum (Fig 2B). After mapping the RA and the CS, the ablation catheter was positioned in the SVC with a septal orientation. During a pullback maneuver, the catheter was observed for the typical drop into the region of the fossa ovalis. The catheter was then advanced through the PFO into the LA (allowing a maximal contact force of 60 g when using the contact force-sensing catheter). The ablation catheter was positioned in the LA and the transseptal sheath was then advanced until the proximal electrodes of the ablation catheter switched from a grey to a black color indicating their position within the sheath and thereby confirming a LA position of the transseptal sheath (Fig 3A). The ablation catheter was then exchanged for a circular mapping catheter (Lasso Nav Variable 2515, Biosense Webster). The LA was then mapped with the Lasso catheter using the FAM feature as described before [3]. The location of the PFO was marked by pulling the Lasso catheter into the sheath and advancing it back into the LA using the FAM feature as described before [9] Then, after pulling the transseptal sheath back to the RA but leaving the Lasso in the LA, the mapping catheter was inserted into the second transseptal sheath and advanced into the RA and through the PFO. Mapping of the inferior left atrium (along the coronary sinus to the inferior septum) and the mitral annulus region was performed with the ablation catheter to prevent entrapment of the Lasso catheter in the mitral valve apparatus (Fig 3B) [3].

After completing the LA map, PVI was achieved by performing wide antral circumferential ablation using radiofrequency energy with the power set to 25–30 Watts and a maximum temperature of 50°C using continuous encircling lesions.

### Outcome measures

The primary outcome measures of the study were the feasibility of fluoroscopy-free PVI in patients with AF and a PFO, total fluoroscopy time (requirement for fluoroscopy verification), total dose area product (DAP), and total procedure time. The use of any radiation during the procedure was counted as a failure.

Secondary outcome measures were complications, acute and chronic success rates of PVI (single procedure), and procedural details (duration of RA/LA mapping, time needed for ablation, net RF time). All complications were classified according to the HRS/EHAR/ECAS expert consensus statement on AF ablation [2].

### Post-ablation management

Transthoracic echocardiography was performed after the procedure to rule out pericardial effusion. Oral anticoagulation was continued for at least 3 months. Antiarrhythmic drugs were discontinued after the procedure. Follow-up consisted of outpatient clinic visits at 3 and 6 months and then every 6 months and included a detailed history, physical examination, 12-lead ECG, 24-hour Holter monitoring and a 7-day Holter at 12 months. In addition, patients were seen in case of recurrent symptoms. Episodes of AF (>30 seconds) were counted as recurrences. Recurrence rates were analyzed with a post-procedural blanking period of three months.

### Statistical analysis

Continuous variables are presented as mean ± one standard deviation or as median and interquartile range (IQR) in case of skewed distribution. For continuous variables, comparisons were made using Student’s T-test, or Mann-Whitney U test, as appropriate. Discrete variables were compared using Fisher’s exact test. A p-value <0.05 was considered to indicate statistical significance. Calculations were made using the SPSS (version 22.0, SPSS Inc., Chicago, ILL) software package.

## Results

### Study population

A total of 30 patients were studied. Patients had a mean age of 61±12 years, 73% were men. 19 patients (63%) had paroxysmal AF, whereas AF was persistent in 11 patients (37%). Baseline characteristics of the patients are provided in Table 1.

**Table 1:** Baseline characteristics.

Variable	
Male	22 (73)
Age [years]	61±12
BMI [kg/m^2^]	26±4
Paroxysmal AF	19 (63)
Hypertension	12 (40)
CAD	4 (13)
Diabetes	3 (10)
LVEF [%]	64 (53–65)
LA (PLAX) [mm]	38±7

Values are n (%) for categorical and mean ± standard deviation or median (interquartile range) for continuous variables. BMI = body mass index; CAD = coronary artery disease; LVEF = left ventricular ejection fraction; LA = left atrium; PLAX: parasternal long axis.

Based on the findings of transesophageal echocardiography, of the 30 patients with a PFO, 3 patients (10%) had an atrial septal aneurysm, 10 (33%) had a hypermobile atrial septum and 17 (57%) had a normal anatomy of the atrial septum apart from the PFO. The PFOs were classified as small in 15 patients (50%), as medium in 12 (40%), and as large in 3 patients (10%). (Fig 1).

### Primary outcome measures

Completely fluoroscopy-free PVI without any additional technical equipment such as peri-procedural intracardiac or transesophageal echocardiography was feasible in 26/30 (87%) patients. The approach was not feasible in 4/30 patients (13%). Total procedure time was 127±37 minutes. Procedural details are summarized in Table 2. Neither the outcome nor any of the interventional parameters was significantly different in patients with successful fluoroscopy-free PVI compared to interventions requiring fluoroscopy.

**Table 2:** Procedural data.

Variable	
Procedure duration	127±37
Stick-Map RA [min]	12±5
Stick-Map LA [min]	25±9
Map RA [min]	9±4
Map LA [min]	16±5
Map overall [min]	29±10
Ablation [min]	82±32
Net RF duration [sec]	1883±926
Fluoroscopy time [min]	0 (0–0)
Radiation dose [Gy*cm^2^]	0 (0–0)

Values are mean ± standard deviation or median (interquartile range) for continuous variables. RA = right atrium; LA = left atrium; RF = radiofrequency; Stick-Map = time from puncture of groin to start of mapping process.

In 2 of 15 patients (13%) with a small PFO and one of 12 patients (8%) with a medium-size PFO, LA access could not be obtained and in another one of 12 patients (8%) with a medium-sized PFO and an atrial septal aneurysm, LA access was lost at the end of LA mapping after successful initial LA access. LA re-access was not feasible without using fluoroscopy in this patient. In these four patients, in whom the fluoroscopy-free approach was not successfully performed, the total fluoroscopy time was 5±3 min and total dose area product was 14.9±13.4 Gy*cm^2^. In the 3 patients with a large PFO, fluoroscopy-free LA access and PVI was successful.

### Secondary outcome measures

Fluoroscopy-free access to the RA, mapping of the caval veins and RA and cannulation and mapping of the CS was feasible in all 30 patients (100%). Crossing the PFO without fluoroscopy was feasible in 27/30 (90%). The time required for right atrial mapping including transseptal crossing was 9±4 minutes. The time needed for completing the ablation procedure after mapping both atria was 82±32 minutes, net RF time was 1883±926 seconds.

The procedural endpoint of PVI documented by a circular mapping catheter was reached in all patients and no complications occurred. Single-procedure success rate was 80% (24/30) after a median follow-up of 12 months.

A contact force sensing-catheter was used in 17 of the 30 patients (57%). No statistical difference in procedural parameters, safety and outcome between procedures performed with and without force-sensing catheters could be observed.

## Discussion

### Main findings

The main findings of this study are: 1) In patients in whom no transseptal puncture is necessary to access the LA due to the presence of an echocardiographically documented PFO, performing PVI completely fluoroscopy-free is feasible in the vast majority of cases. Our approach was feasible without the intra-procedural use of intracardiac or transesophageal echocardiography 2) The approach was not feasible in a minority of 13% of patients; however, even in case of failure, total fluoroscopy time and radiation dose were very low. 3) There were no complications associated with fluoroscopy-free PVI.

### Reduction of radiation exposure

The use of ionizing radiation is associated with acute (e.g. radiation dermatitis) and chronic (e.g. neoplasms) negative effects that should ideally be outweighed by the benefit of the information gained by the result of radiological exams [10]. To some extent, catheter ablation of AF and other arrhythmias is still performed under fluoroscopic guidance [11, 12], but the advent of EAM systems has reduced the radiation burden dramatically, not only for the patient, but also the physician and the lab staff. Since fluoroscopy duration is, beside the type and configuration of the X-ray system, the collimation, the angulations and the patient characteristics, directly linked to DAP, reduction of fluoroscopy duration results in a reduction of ionizing radiation.

The defining element of the present study is that performing PVI was feasible by using a standardized approach with a latest-generation mapping system, a pre-procedural MRI, but no additional technical equipment. However, to be able to perform fluoroscopy-free PVI in 100% of patients with a documented PFO, TEE or ICE to gain access to the LA would be needed in a minority of patients.

Our approach should be considered especially for younger patients with a higher lifetime risk of radiation-induced neoplastic disease. The herein presented data are in the same range as in our recent study investigating PVI without fluoroscopic restrictions in a similar patient cohort [3]. In that study, we reported a success rate of 72% and a procedural duration of approximately 130 minutes compared to a success rate of 80% and procedure duration of 127 minutes in the current study. Therefore, the fluoroscopy-free approach does not appear to come at the expense of longer procedure times and more arrhythmia recurrences.

### Technical considerations and workflow

The feasibility of fluoroscopy-free mapping and ablation in the LA (after fluoroscopically-guided transseptal puncture) using the latest generation EAM system has recently been reported.^3^ At the same time, a zero-fluoroscopy approach has been reported for arrhythmias originating in the RA using EAM systems [13, 14]. Our study is the first report of a completely fluoroscopy-free PVI without additional technical equipment such as peri-procedural intracardiac or transesophageal echocardiography. Although not deemed indispensible, the use of a contact force-sensing catheter may further enhance the safety during different steps of the procedure (e.g. reaching the RA without fluoroscopy, mapping the RA, passing through the PFO) in order to avoid the consequences of applying excessive force. However, it is unknown whether there is a specific contact force cut-off that should be avoided in different regions to prevent complications. Fluoroscopy-free PVI requires following a structured procedural workflow. We acknowledge that the acquisition of the required map of the RA adds time to the total procedure duration, but the time needed for this is limited (<10 minutes). Therefore, there is no relevant increase in the complexity of the procedure when following the proposed procedural protocol. Cardiac MRI with a focus not only on the left atrial but also right atrial anatomy could potentially decrease the need for accurate right atrial mapping.

### Echocardiographic evaluation

All three patients in whom the LA could not be accessed through the PFO had a PFO that was classified as “small” based on echocardiography. This suggests that PFOs classified as “medium” or “large” may usually be passed without the use of fluoroscopy. In the one patient in whom LA re-access was not feasible, there was an echocardiographically documented atrial septal aneurysm. It is conceivable that the presence of an aneurysmatic sac can prevent the catheter from being advanced through the PFO in regular fashion. It remains unclear why initial LA access was feasible but re-access failed. It should be noted that the current study is too small to determine predictors of failure of the fluoroscopy-free approach.

### Risks

Although no complications occurred in this series, entry into the pericardium is a potential risk when using the PFO to access the LA. However, this also holds true when using a fluoroscopic approach. Lehrmann et al. showed that interatrial septum dissection and perforation into the pericardial space may occur when probing the septum to gain access to the LA [15]. In our study, we only attempted to cross the septum in case of an echocardiographically documented PFO. Furthermore, the use of a contact force-sensing catheter may be helpful in avoiding high forces when probing the PFO. Also, monitoring contact-force and its vector orientation when advancing the catheter from the venous access site to the RA may enhance the safety of the fluoroscopy-free procedure.

### Limitations

This is a non-randomized single center study. Due to the sample size, no definite conclusions can be drawn with regards to the safety of the reported approach based on this study. Because of the prevalence of the PFO, the reported approach may only be used in approximately one quarter of all patients referred for PVI. Transesophageal echocardiography was performed with propofol sedation. This may result in underestimation of the true PFO prevalence and impact the classification of the PFO size despite performing a passive Valsalva maneuver. Strict exclusion of fluoroscopy may actually increase the risk of procedural complications to a patient especially if the operator has a preconceived reluctance to avoid fluoroscopy in an effort to promote positive results of this trial. However, this was not seen in our study.

## Conclusion

This study demonstrates that by using features of a latest generation EAM system in conjunction with a high-quality pre-procedural MRI and a standardized protocol, fluoroscopy-free PVI is feasible in patients with a PFO without additional technology such as peri-procedural intracardiac or transesophageal echocardiography. While being especially attractive for younger patients or even pregnant women, a fluoroscopy-free approach to PVI may be considered in any patient with a documented PFO. In order to reduce the radiation burden to patients, physicians and staff in the electrophysiology laboratory, the described approach could potentially be adapted for a vast majority of ablation procedures.

## Supporting Information

S1 Data File of the complete data. Abbreviations according to manuscript.
(XLSX)
